# Formulation Development and Investigations on Therapeutic Potential of Nanogel from *Beta vulgaris* L. Extract in Testosterone-Induced Alopecia

**DOI:** 10.1155/2023/1777631

**Published:** 2023-01-31

**Authors:** Sumitra Singh, Sonia, Rakesh K. Sindhu, Abdulrahman A. Alsayegh, Gaber El-Saber Batiha, Saqer S. Alotaibi, Sarah M. Albogami, Carlos Adam Conte-Junior

**Affiliations:** ^1^Department of Pharmaceutical Sciences, Guru Jambheshwar University of Sciences and Technology, Hisar, Haryana 125001, India; ^2^School of Pharmacy, Sharda University, Greater Noida, Gautam Buddha Nagar, Uttar Pradesh 201306, India; ^3^Clinical Nutrition Department Applied Medical Sciences College, Jazan University, Jazan 8281, Saudi Arabia; ^4^Department of Pharmacology and Therapeutics, Faculty of Veterinary Medicine, Damanhour University, Damanhour, Egypt; ^5^Department of Biotechnology, College of Science, Taif University, P.O. 11099, Taif 21944, Saudi Arabia; ^6^Center for Food Analysis (NAL), Technological Development Support Laboratory (LADETEC), Federal University of Rio de Janeiro (UFRJ), Cidade Universitaria, Rio de Janeiro 21941-598, Brazil

## Abstract

The objective of the present study was to develop a novel nanogel containing *Beta vulgaris* L. hydroalcoholic extract and assess its efficacy for treating testosterone-induced alopecia. *Beta vulgaris* L. leaf hydroalcoholic extract nanogel (BVEN) was prepared by ionic gelation method, incorporated in carbopol 934 gel. Optimization of particle size and entrapment efficiency as the responses was carried out by central composite design response surface methodology. Prepared nanoparticles were evaluated for entrapment efficiency, particle size, zeta potential, polydispersity index, Fourier transform infrared spectroscopy, transmission electron microscopy, and differential scanning calorimetry. Nanogel was evaluated for pH, colour, appearance and homogeneity, viscosity, spreadability, *in vitro* release study, and stability studies. Further, 2.5% and 5% BVEN were also evaluated for antialopecic activity in Swiss albino mice by using parameters as hair growth initiation, testosterone content, total protein, prostate weight measurement, hair follicular density, anagen/telogen ratio, and histopathological studies. The resulting nanoparticles had better entrapment efficiency with particle size of 274 nm, polydispersity index of 0.259, and zeta potential of +28.8. BVEN pH 6.5, drug content, i.e., quercetin 99.84 ± 1.30% and stigmasterol 99.89 ± 1.52%, spreadability 20.3 ± 0.5925 g cm/sec, and viscosity 110 × 10^5^ cps were observed. Stability studies showed that nanogel was stable at 4°C ± 2°C/60% ± 5% RH. It was found that 5% BVEN showed better antialopecic activity as compared to 2.5% BVEN.

## 1. Introduction

The novel drug delivery systems and nanotechnology have proven to increase the chances of herbal-based therapies being implemented due to boosting the potential of medication action, promoting the sustained release of active ingredients, reducing the required dosage, and improving biological activity [1–3]. A nanogel is a nanoparticle that contains a hydrogel with a polymer network that is cross-connected [4–8]. Diffusion, degradation, pH, and environmental stimulation affect phytochemical release from nanogels [3, 9]. Polymeric nanoparticles, solid lipid nanoparticles, lipid crystal systems, liposomes, nanogels, nanotubes, and nanoemulsions have all been tried as carrier vehicles to preserve herbal medications from external degradation while also increasing their bioavailability [3, 7, 8, 10, 11]. *Beta vulgaris* L. (family Chenopodiaceae), a herbaceous biennial or, rarely, perennial plant, is native plant to Mediterranean regions [12, 13]. Its leaves contain various phytoconstituents like flavonoids like quercetin, polyphenols, betalains, vitamins, minerals, stigmasterol, and *β*-sitosterol [11, 14–17]. In addition to being an expectorant and diuretic, beet roots are also used as a natural food dye in dairy and meat products as well as a treatment for liver and mental health diseases. The leaves of *B. vulgaris* possess diuretic, purgative, anti-inflammatory, wound healing, hepatoprotective, *in vivo* antioxidant, antiparkinson, antidiabetic, and antianxiety activities [17].

In this paper, a novel BVEN was developed. Prepared and optimized nanoparticles comprising alginate and chitosan polymers were added to carbopol 934 gel to formulate BVEN. Then, nanoparticles and BVEN were evaluated by different techniques. Testosterone-induced alopecia is a heritable, androgen-dependent disorder that occurs in a defined pattern characterized by the structural miniaturization of androgen-sensitive hair follicles [18]. Testosterone is the major circulating androgen which is converted to 5-*α*-dihydrotestosterone (5-*α*-DHT) by the 5-*α*-reductase-II enzyme. Hair follicles are the targets for androgen-stimulated hair follicle miniaturization, leading to replacement of large, pigmented hairs known as terminal hairs by barely visible, depigmented hairs called as vellus hairs [19]. As a result, the density of the visible scalp hair gradually decreases. The main treatments of it include hair transplant surgery, wigs and hairpieces, oral medicines like finasteride and minoxidil, hormone action modifiers, nonhormonal therapy, and laser irradiation, although no treatment till date can fully cure this disease and also drugs associated with side effects [20].

Hence, to treat testosterone-induced alopecia and to minimize the side effect of drugs, novel strategies are needed. Preliminary phytochemical study confirmed the presence of carbohydrates, alkaloids, flavonoids, tannins and phenolic compounds, saponins, steroid and triterpenoids, and fatty acids in hydroalcoholic extract of *B. vulgaris* leaves [17]. Phytoconstituents like fatty acids, tannins, phenolic acids, flavonoids (quercetin), phytosterols might be responsible for inhibition of *α*-reductase-II [21, 22], finally, antigen stages are triggered and testosterone conversion to dihydrotestosterone is inhibited, resulting in hair growth.

BVEN containing nanoparticles provide greater bioavailability of the drug at the site of action because their smaller diameter gets arrested in the hair follicle structure [23]. Thus, novel BVEN was formulated and biologically evaluated in Swiss albino mice.

## 2. Materials and Methods

### 2.1. Plant Materials

*Beta vulgaris* leaves were collected from fields of Haryana in the month of December. The plant was authenticated by Dr. Anjula Pandey, principal scientist, National Herbarium of Cultivated Plants, NBPGR, New Delhi, vide reference no. NHCP/NBPGR/2017-22, dated 03.01.2017. The plant was identified as *Beta vulgaris* L. family Chenopodiaceae.

### 2.2. Chemicals

Chitosan (low molecular weight), sodium alginate, calcium chloride, acetic acid, sodium hydroxide, carbopol 934, methyl paraben, propyl paraben, propylene glycol, triethanolamine, finasteride (all were procured from Hi-Media laboratories, Mumbai, India), testosterone (Loba Chemie Pvt Ltd., Mumbai, India), and carboxymethylcellulose (Sigma-Aldrich) were used. All chemicals and solvents used were of analytical reagent grade.

#### 2.2.1. Preparation of Drugs and Solutions

The standard drug finasteride was prepared in ethanol/propylene glycol/water (8 : 1 : 1). Testosterone solution was prepared as 1% *w*/*v* suspension in aqueous carboxyl methyl cellulose solution [21].

### 2.3. Quantification of Quercetin and Stigmasterol by High Performance Liquid Chromatography (HPLC)

HPLC was used to determine the quantity of stigmasterol and quercetin at 258 nm and 206 nm, respectively. The experiments were carried out on a Shimadzu Prominence series HPLC system, which included a solvent delivery unit (model LC-20AD), a variable wavelength UV/VIS detector (model SPD-20A), and a Hamilton manual injection valve with a 20 *μ*l loop, C18 column (250 × 4.6 mm, 5 m i.d.), and degasser (Aczet, Helix Biosciences). LC solution software was used for data collection and analysis. Methanol/0.1 percent formic acid (65 : 35 *v*/*v*) was employed as the mobile phase for quercetin, while methanol was used for stigmasterol. Calibration curve was prepared by using standard quercetin and stigmasterol.

### 2.4. Preparation of the Reference Solution

Standard stock solution (1 mg/ml) was prepared by dissolving quercetin and stigmasterol in methanol. Further dilutions were prepared from stock solution. Solutions were filtered through a membrane filter of 0.22 *μ*m and sonicated.

### 2.5. Preparation of Sample Solution

Hydroalcoholic extracts 5 mg/ml and 10 mg/ml were dissolved in methanol for estimation of quercetin and stigmasterol, respectively. Then, sample solution was filtered through a membrane filter of 0.22 *μ*m and sonicated [24, 25].

### 2.6. Preparation of Nanoparticles

The hydroalcoholic extract of *B. vulgaris* L. leaves was prepared [17], formulated as nanoparticles, and optimized. To make nanogel, the nanoparticles were incorporated into gel. Ionic gelation method was used to make nanoparticles [26, 27]. Chitosan stock solution was prepared by dissolving 500 mg chitosan in 50 ml of 1% *v*/*v* acetic acid solution. 50 ml distilled water each was used to dissolve 500 mg sodium alginate and 500 mg calcium chloride [28]. 0.6 ml of chitosan stock solution was further diluted to make a 10 ml solution, with 1% *v*/*v* acetic acid solution. Sodium alginate and calcium chloride were both optimized. Sodium alginate and calcium chloride stock solutions were prepared by diluting with 10 ml and 1 ml of distilled water, respectively. Dropwise diluted 1 ml calcium chloride was added to the diluted 10 ml sodium alginate while stirring to make a pregel. While stirring, 5 mg/ml hydroalcoholic extract was added dropwise to 10 ml diluted chitosan solution, and the produced solution was then added dropwise to calcium alginate pregel. 0.1 N sodium hydroxide was used to adjust the pH to 5.3, and the mixture was agitated for another 30 minutes. The sample was centrifuged for 10 minutes at 1100 rpm to remove any big aggregates in the form of pellets, leaving nanoparticles in the supernatant. To separate free polymer from nanoparticles, the particle suspension was centrifuged at 5000 rpm and 25°C temperature for 20 minutes. This solution was used to do further research. Also, nanoparticle solution was lyophilized for analysis. Nanoparticles without extract (blank) were also prepared in similar way for comparison [26].

### 2.7. Preparation of Nanogel

In a beaker, 1 g carbopol 934 was spread in 50 ml distilled water. After allowing the carbopol to swell for half an hour, stir for 30 minutes with a mechanical stirrer at 1200 rpm [29]. In a separate beaker, put 5 ml propylene glycol, weigh amounts of propyl paraben and methyl paraben, and mix well. After all, steady stirring was used to add carbopol dispersion, lyophilized nanoparticles, and preservative solutions. Finally, the volume was increased to 100 ml by adding the remaining distilled water, and triethanolamine was added drop by drop to get the appropriate skin pH (6.8-7) and gel consistency [30].

For the optimization of nanoparticles, Design-Expert software version 10.0.6.0 (Stat-Ease, Inc., Minneapolis, MN, USA) was used for generation of polynomial equations with extra interaction terms for the correlation of selected answers with selected factors. For systemic optimization, particle size and entrapment efficiency were chosen as response variables. The software also supplied the best answer by using overlay plots. The face-centered central composite design was used to determine the optimum concentration of sodium alginate and calcium chloride. The effect of two components, namely, the amount of sodium alginate and the amount of calcium chloride, on two response variables, namely, entrapment efficiency (R1) and particle size, was examined (R2). Factor levels were appropriately coded. Experiments (13 runs) were carried out with various levels of the chosen components (Table 1).

### 2.8. Evaluation of Optimized Nanoparticles

Different parameters were studied to optimize nanoparticles [26, 31].

#### 2.8.1. Entrapment Efficiency

Nanoparticles were removed from suspension by ultracentrifugation at 10,000 rpm and 40°C for 30 minutes after entrapment efficiency was determined. The following equation was used to compute the entrapment efficiency: Entrapment efficiency = *T* − *F*/*T* × 100 where *T* is the total amount of medication in the supernatant and *F* is the free amount of medication that was added to the chitosan solution.

#### 2.8.2. Particle Size Determination, Polydispersity Index, and Zeta Potential

The nanoparticles were suitably diluted in distilled water for measurements of particle size, polydispersity index, and zeta potential on zeta sizer in automatic mode. Instrument was operated at 25°C temperature and a scattering angle of 90°. To determine the long-term physical stability of the colloidal systems, the zeta potential was evaluated. Polydispersity is a measure of particle homogeneity and it varies from 0 to 1. The nearer the value of polydispersity to zero, the higher is the homology between the particles [32]. Nanoparticles with zeta potential values greater than +25 mV or less than -25 mV typically have high degrees of physical stability [33, 34]. Characterization of all three properties was studied in triplicate.

#### 2.8.3. Fourier Transform Infrared Spectroscopy (FTIR)

FTIR instrument of Spectrum RX, Perkin Elmer, USA, was used to check any chemical interaction of drug and excipients in the nanoparticles. Lyophilized nanoparticles were used for FTIR analyses. The KBr pellets of extracts, excipients, blank nanoformulation, and optimized nanoformulations were scanned in the frequency range 4000-400 cm^−1^.

#### 2.8.4. Transmission Electron Microscopy (TEM)

High-resolution TEM of Tecnai G20FEI Inc., USA, was used for the analysis of optimized batch. Nanoparticles were diluted with HPLC grade water and sonicated for 4 minutes to disunite nanoparticles. The samples were placed on a carbon-covered grid (300 mesh) and dyed with 2% phosphotungstic acid (pH 7.0) before being examined at 200 kV.

#### 2.8.5. Differential Scanning Colorimetry (DSC)

DSC instrument (TA Instruments DSC SDT Q600) was operated under dry nitrogen (flow rate 100 ml/min) atmosphere bearing temperature range of 30°C to 400°C with a heat flow rate of 100°C/min. For analyzing the data, Universal Analysis 2000 software (SDT Instruments) was used [35].

#### 2.8.6. Evaluation of Nanogel

pH, physical appearance, viscosity, drug content, spreadability, *in vitro* release tests, and stability studies were all carried out in accordance with ICH criteria [36, 37]. The viscosity of BVEN was tested using a Brookfield viscometer with spindle 6. Formulations were filled into scintillation glass vials and incubated for three months at 4°C ± 2°C/60% ± 5% RH, 25°C ± 2°C/65% ± 5% RH, and 40°C ± 2°C/75% ± 5% RH, as per ICH guidelines. pH, physical appearance, viscosity, drug content, spreadability, etc. were all performed in triplicate [29, 38].

#### 2.8.7. *In Vitro* Release Study

The nanogel release was studied *in vitro* using the Franz diffusion cell. The goal of the investigation was to see how much medication was released from the nanogel. The membrane employed was a porous membrane with a molecular weight cutoff of 50,000 Da (Sigma-Aldrich). The membrane was installed between the instrument's donor and receiver compartments. The donor compartment was filled with nanogel, while the receiver compartment was filled with 10 ml of phosphate buffer (pH 7.4), swirled, and kept at 37°C ± 0.5°C in a circulator water bath. Paraffin paper was used to cover the top. 0.1 ml of sample was withdrawn from the receiver compartment at specified intervals (1, 2, 4, 6, 8, and 10 hours) and replaced with fresh buffer solution [39]. The quercetin and stigmasterol contents of the obtained samples were determined. To determine average percent release, the cumulative amount of drug permeated through the skin was calculated [37].

A curve shown between time taken on *X* axis and percentage cumulative drug release on *Y* axis is known as zero-order plot. A curve shown between time taken on *X* axis and log percentage cumulative drug release on *Y* axis is known as first-order plot. The Higuchi plot is known as a curve drawn between square root of time taken on *X* axis and cumulative drug release on *Y* axis. In the Korsmeyer-Peppas plot, a curve was drawn between log time taken on *X* axis and log percentage cumulative drug release on *Y* axis [40, 41].

### 2.9. *In Vivo* Testosterone-Induced Alopecia Activity

#### 2.9.1. Experimental Animals

Twenty-four male Swiss albino mice (20-25 g in weight), 3-4 months old, were obtained from disease-free small animal house, Lala Lajpat Rai University of Veterinary and Animal Sciences, Hisar, India. The experimental protocol was approved by the Institutional Animal Ethics Committee for the purpose of control and supervision of experiments on animals, Ministry of Environment and Forests, Government of India (registration no. CPCSEA/436/PO/Re/S/2001, dated 20.06.2016). For the investigations, the experimental animals were maintained under typical environmental conditions of temperature and humidity (25 ± 0.5°C) and a 12-hour light/dark cycle. Pellet feed and water were given to the animals.

*(1) Grouping of Animals*. Group 1 (control): animals treated with testosterone and blank nanogel (topical)

Group 2 (standard): animals treated with testosterone and 2% finasteride (0.2 ml, topical)

Group 3 (test): animals treated with testosterone and 2.5% BVEN (topical)

Group 4 (test): animals treated with testosterone and 5% BVEN (topical)

*(2) Dose Selection*. The doses of testosterone solution (0.5 mg/ml/day, s.c. for induction of alopecia), 2% finasteride, BVEN (2.5% and 5% solution of formulations) were used [42, 43].

#### 2.9.2. Experimental Protocol

Noubarani et al. [44] described an approach that was followed with minor modifications. Swiss albino mice were split into four groups, each with six animals. Hairs from a 3 cm^2^ area on the dorsal portion of all mice were shaved using electric shavers and removed entirely with a commercial hair remover [45]. Finally, surgical spirit was used to wipe off the denuded skin. All groups of mice received a testosterone dosage (0.5 mg/ml/day, s.c.). For 30 days, animals were administered different topically applied treatments on their dorsal skin surface.

#### 2.9.3. Quantitative Study

After 10th day, mouse skin was observed for hair growth initiation with magnifying lens. Visual observations and photographs were used to document the differences in hair development in each group up to the 30th day of the trial. On the 30th day, blood samples were collected for testosterone content measurement and total protein content. Also, mice were selected and sacrificed from each group for prostate gland weight measurement.

#### 2.9.4. Qualitative Study

On the 20th and 30th days, mice from each group were randomly chosen for a skin biopsy from the balding site, and the samples were stored in phosphate-buffered formalin for paraffin sectioning. Hematoxylin and eosin were used to stain vertical sections (3–4 *μ*m) cut parallel to the direction of hair development. With the use of an ocular micrometre, the cyclic phase of hair follicles (anagen and telogen) and follicular density were assessed. A skin biopsy was collected from the balding area [43, 46].

#### 2.9.5. Statistical Analysis

All results are expressed as mean ± SEM. Results were analyzed by one-way analysis of variance (ANOVA) using Dunnett's test where ^∗^*p* < 0.05, ^∗∗^*p* < 0.01, and ^∗∗∗^*p* < 0.001, significance versus group 1 (control). GraphPad InStat software (San Diego, USA) was used for statistical analysis.

## 3. Results and Discussions

### 3.1. Quantification of Phytoconstituents in *B. vulgaris* Hydroalcoholic Extract

**Figure 1:**
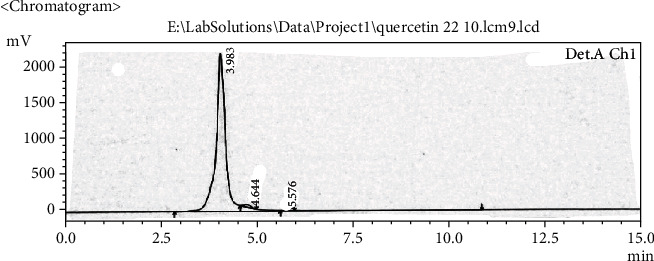
Chromatogram of standard quercetin.

**Figure 2:**
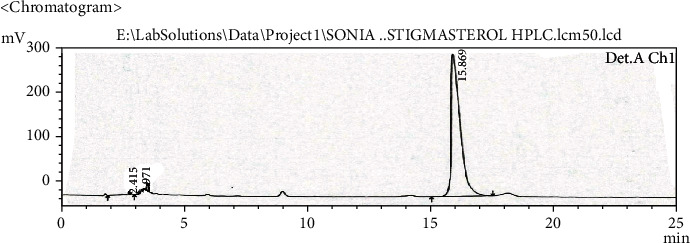
Chromatogram of standard stigmasterol.

**Figure 3:**
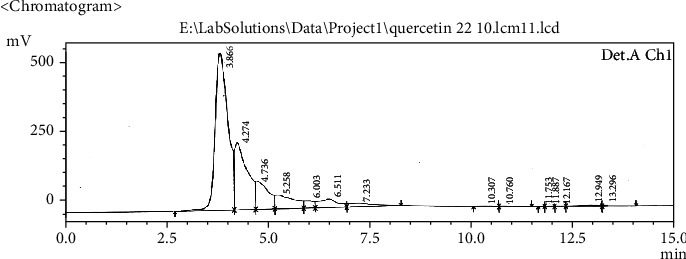
Chromatograms of *B. vulgaris* hydroalcoholic extract for quercetin.

**Figure 4:**
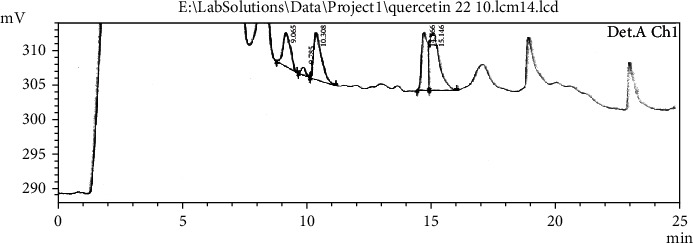
Chromatograms of *B. vulgaris* hydroalcoholic extract for stigmasterol.

Extract solution was analyzed to identify and quantify the quercetin and stigmasterol. HPLC chromatogram (Figures 1–4) for standard quercetin, stigmasterol, and extract was recorded, and peak area was determined in triplicate using calibration curve equation. Quercetin was found 18.97 *μ*g/mg and stigmasterol 2.45 *μ*g/mg.

### 3.2. Optimization of Nanoparticles Containing *Beta vulgaris* L. Leaf Hydroalcoholic Extract by Central Composite Design

Based on entrapment efficiency and particle size measurements, a face-centered central composite design was employed to determine the optimum concentration of sodium alginate and calcium chloride. Thirteen formulations were made, and the effect of two components, sodium alginate (A) and calcium chloride (B), on the two response variables, drug entrapment efficiency (DEE) and particle size, was examined. The average particle size for all batches was between 247 and 712 nm. Drug entrapment efficiency ranges from 56 to 85% for *B. vulgaris* hydroalcoholic extract containing quercetin while for stigmasterol 43-79%.

**Figure 5:**
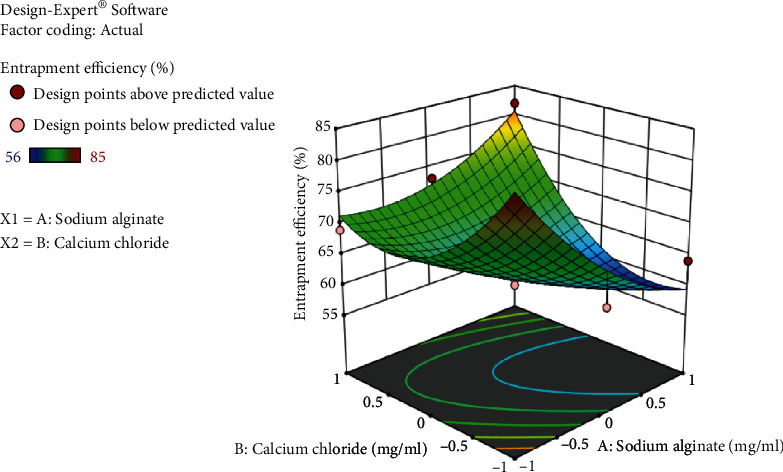
3D response surface graph showing effect of sodium alginate and calcium chloride concentration on entrapment efficiency of *B. vulgaris* extract for quercetin nanoparticles.

**Figure 6:**
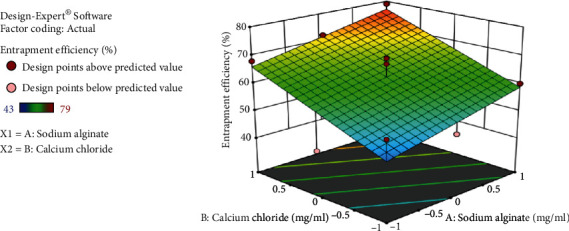
3D response surface graph showing effect of sodium alginate and calcium chloride concentration on drug entrapment efficiency of *B. vulgaris* extract for stigmasterol nanoparticles.

Tables 2 and 3 demonstrate the optimal parameters for optimized formulation as proposed by Design-Expert software, which resulted in the formation of nanoparticles. DEE of *B. vulgaris* extract for quercetin and stigmasterol nanoparticles is shown in Figures 5 and 6, respectively, presenting 3D response surface graph which reflects the concentration effect of sodium alginate and calcium chloride on. For the responses (DEE and particle size), the polynomial equation produced by regression analysis is as follows (Yadav et al., [18]):

(1) $$\begin{array}{c} \text{DEE of quercetin}:+61.86-3.83*\text{A}+2.17*\text{B}+8.50*\text{AB}+3.98*\text{A }2+7.98*{\text{B}}^{2}. \end{array}$$

In the above equation, the positive and negative symbols before coefficients represent their synergistic or antagonistic effect on the responses, respectively. Equation (1) indicated that the maximum entrapment efficiency was achieved with decreased concentration of sodium alginate and increased concentration of calcium chloride.

(2) $$\begin{array}{c} \text{DEE of stigmasterol}:+62.31+5.5*\text{A}+9.17*\text{B}. \end{array}$$

Entrapment efficiency of stigmasterol increases with increase in concentration of sodium alginate and calcium chloride shown in equation (2).

(3) $$\begin{array}{c} \text{Particle size}:+447.29-32.02*\text{A}-45.2*\text{B}-157.3*\text{AB}+988\text{A }2-15.55*{\text{B}}^{2}. \end{array}$$

**Figure 7:**
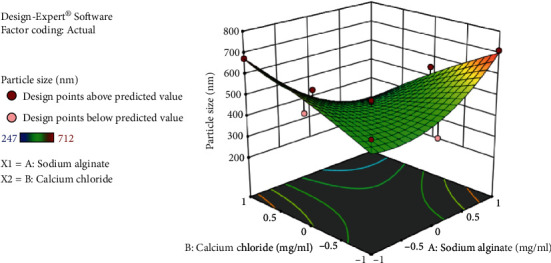
3D response surface graph showing effect of sodium alginate and calcium chloride concentration on particle size of *B. vulgaris* extract nanoparticles.

Figure 7 shows a 3D response surface graph depicting the effect of sodium alginate and calcium chloride concentrations on particle size. Particle size plays an important role in nanoparticles; the smaller the size, the deeper would be the penetration. Particle size decreases with increase in concentration of sodium alginate and calcium chloride as shown in equation (3).

#### 3.2.1. Entrapment Efficiency

Entrapment efficiency of *B. vulgaris* hydroalcoholic extract-optimized nanoparticles for quercetin and stigmasterol was performed. Drug entrapment efficiency for quercetin and stigmasterol was 82% and 79%, respectively.

#### 3.2.2. Particle Size Determination, Polydispersity Index, and Zeta Potential

**Figure 8:**
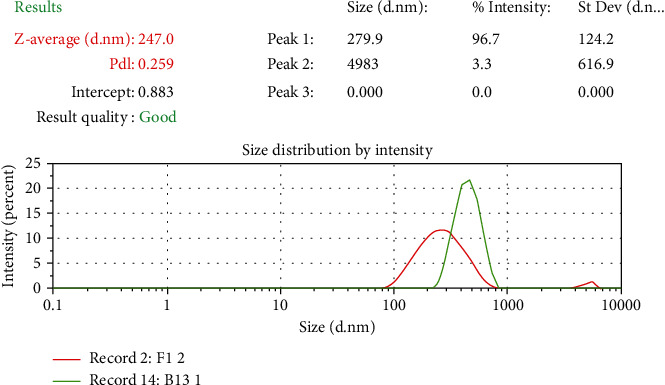
Particle size and polydispersity index of optimized nanoparticles containing *B. vulgaris* hydroalcoholic extract.

**Figure 9:**
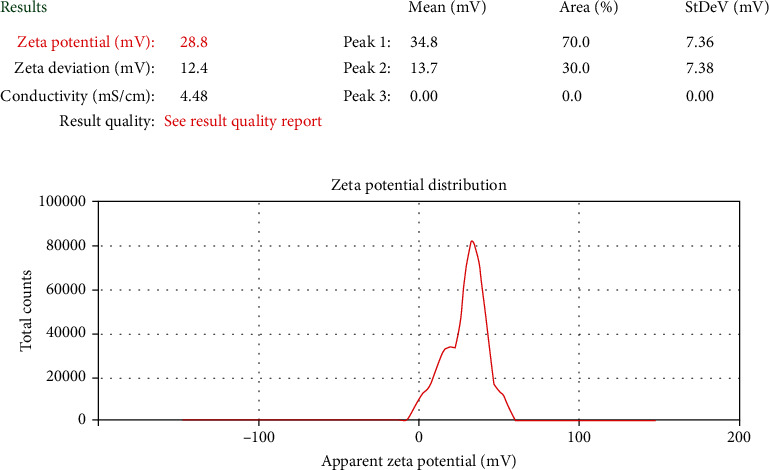
Zeta potential of optimized nanoparticles containing *B. vulgaris* hydroalcoholic extract.

Particle size of *B. vulgaris* hydroalcoholic extract-optimized nanoparticles was studied on zeta sizer. The polydispersity index ranging from 0 to 1 indicates wide particle size distributions. These results suggest that nanoparticles were formed with more homogenous size distributions due to the size reduction. Particle size of *B. vulgaris* hydroalcoholic extract-optimized nanoparticles was observed to be 247 nm (Figure 8). Polydispersity index of *B. vulgaris* hydroalcoholic extract-optimized nanoparticles was observed to be 0.259 (Figure 8). The nanoparticles had the higher zeta potential value, indicating the better stability of this colloidal system of particles. This resulted from the electrostatic repulsion, which could prevent the biocolloids from aggregation of nanoparticles. Zeta potential was found to be +28.8 mV (Figure 9) which indicates that nanoparticles were physically stable.

#### 3.2.3. Fourier Transform Infrared Spectroscopy (FTIR)

**Figure 10:**
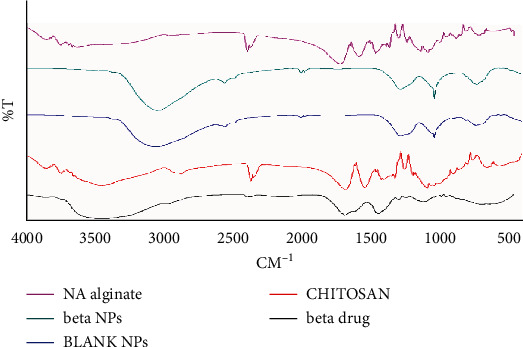
FTIR analysis of optimized nanoparticles containing *B. vulgaris* hydroalcoholic extract.

FTIR was adopted to characterize the potential interactions between components of nanoparticle systems (Figure 10).

Polyelectrolyte interactions were taken into consideration. It has long been known that the anionic polymer's carboxyl group can interact with the amino group of chitosan to generate an ionic complex. The broad band at 3447 cm^−1^ corresponded to hydroxyl and amine groups, the peak at 2866 cm^−1^ was a result of -OH group stretching, the absorption band of the carbonyl (C=O) stretching of the secondary amide (amide I band) is represented by the peak at 1676 cm^−1^, and the bending vibrations of the N-H (N acetylated residues and amide II band) are represented by the peak at 1539 cm^−1^, according to FTIR spectra of chitosan. The N-H stretching of the amide and ether bonds, as well as N-H stretching (amide III band), is represented by the peaks at 1418 and 1339 cm^−1^, respectively. The secondary hydroxyl group (characteristic peak of -CH-OH in cyclic alcohols and C-O stretch) and the primary hydroxyl group were found at 1085 and 1033 cm^−1^, respectively (characteristic peak of -CH2OH in primary alcohols and C-O stretch).

The stretching vibrations of the hydroxyl group were represented by spectral bands at 3649 cm^−1^ and 3618 cm^−1^ in sodium alginate. Asymmetric and symmetric stretching peaks of carboxylate salt groups were attributed to the bands at 1681 and 1418 cm^−1^, respectively. Alginate saccharide structure is responsible for the band at 1032 cm^−1^ (C-O-C stretching).

The asymmetrical stretching of -COO- groups has shifted to 1635 cm^−1^, whereas the symmetrical stretching of -COO- groups has shifted to 1415 cm^−1^ in the FTIR spectrum of nanoparticles of *B. vulgaris* hydroalcoholic extract. Chitosan's stretching vibration of -OH and -NH_2_ at 3447 cm^−1^ moves to 3435,3430,3436 cm^−1^ and becomes broad following interaction with alginate. These findings suggest that sodium alginate carboxylic groups interact with chitosan ammonium groups via electrostatic interactions to produce a polyelectrolyte complex [47, 48].

#### 3.2.4. Transmission Electron Microscopy (TEM)

**Figure 11:**
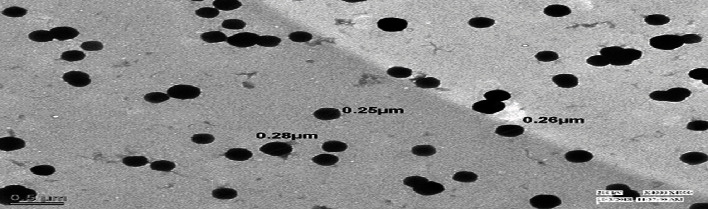
TEM images of optimized nanoparticles containing *B. vulgaris* hydroalcoholic extract.

Results from TEM analysis revealed spherically shaped nanoparticle as shown in Figure 11. Optimized nanoparticle size ranges from 250 nm to 280 nm in *B. vulgaris*.

#### 3.2.5. Differential Scanning Colorimetry (DSC)

**Figure 12:**
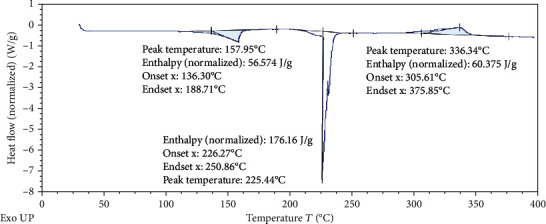
DSC thermogram of *B. vulgaris* hydroalcoholic extract.

**Figure 13:**
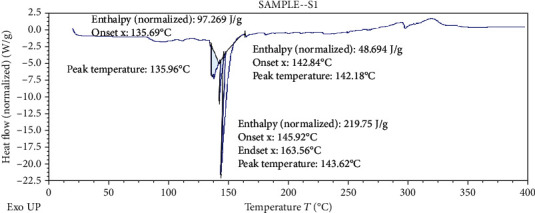
DSC thermogram of blank nanoparticles.

**Figure 14:**
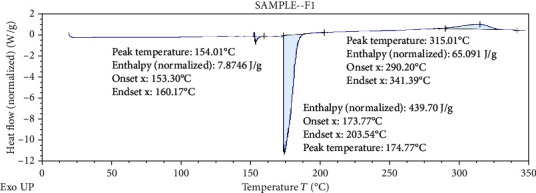
DSC thermogram of optimized nanoparticles containing *B. vulgaris* hydroalcoholic extract.

Differential scanning calorimetry gives the information regarding the crystalline or amorphous nature of the drug using the fact that different drugs and excipients possess different melting points [49]. Differential scanning calorimetry was performed for *B. vulgaris* hydroalcoholic extract, optimized nanoparticles of *B. vulgaris* hydroalcoholic extract, and blank nanoparticles as shown in Figures 12–14. Exothermic peak of alginate and chitosan nanoparticles (blank nanoparticles) was recorded at 143.62°C, which was interpreted as an interaction between both components [26, 49]. DSC of *B. vulgaris* leaf extract exhibited a sharp melting exothermic peak at 225.44°C (Figure 12) that was ascribed to drug melting. Sharp exothermic peaks confirm the crystalline nature of the drug. DSC of blank nanoparticles showed exothermic peak at 143.62°C, and *B. vulgaris* extract-loaded nanoparticles showed exothermic peak at 174.77°C. Shifting of exothermic peak from 225.44°C to 174.77°C could be indicated that *B. vulgaris* extract may have been homogeneously dispersed in the nanoparticles.

#### 3.2.6. Evaluation of Nanogel

Prepared BVEN was evaluated for physical appearance, pH, drug content (quercetin and stigmasterol), spreadability, and viscosity as shown in Table 4. BVEN was light green, smooth, transparent, and pH in range 6.5 (in triplicate). Spreadability was found to be 20.3 ± 0.5925 g cm/sec. Viscosity of nanogel was found to be 110 × 10^5^ cps. Percent of quercetin was found to be 99.84 ± 1.30 and stigmasterol 99.89 ± 1.52 in BVEN nanogel.

### 3.3. Stability Studies

BVEN formulations were studied for stability for up to 3 months at different temperatures 4°C ± 2°C/60 ± 5 RH, 25°C ± 2°C/60 ± 5 RH, and 40°C ± 2°C/65 ± 5 RH. The samples were analyzed for physical appearance, viscosity, pH, drug content, and spreadability. Viscosity, pH, drug content, and spreadability were performed in triplicate. It was observed that BVEN showed no noticeable changes in physical appearance, pH, drug content, spreadability, and viscosity for a period of 90 days at low temperature (4°C ± 2°C/60 ± 5 RH, Table 5); however, at 25°C ± 2°C/60 ± 5 RH, there were slight changes in the physical appearance, pH, drug content, spreadability, and viscosity (Table 6). But at 40°C ± 2°C/65 ± 5 RH, there was much change in physical appearance, pH, drug content, spreadability, and viscosity (Table 7) of all three nanogels. Thus, it was concluded that refrigerated condition (4°C ± 2°C) was more stable storage condition for BVEN formulation.

### 3.4. *In Vitro* Drug Release

**Figure 15:**
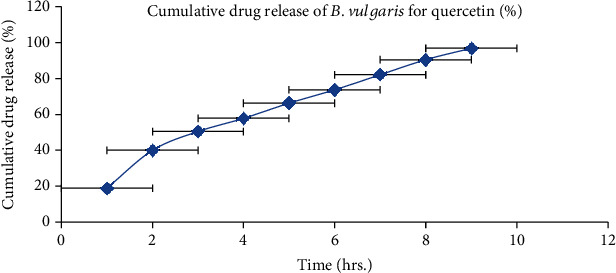
*In vitro* drug release of nanogel containing *B. vulgaris* extract (quercetin). Each value represents the mean ± SEM (*n* = 3).

**Figure 16:**
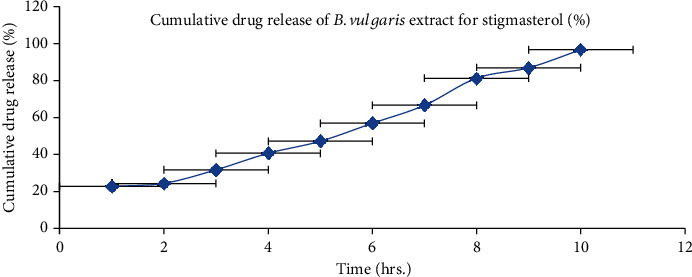
*In vitro* drug release of nanogel containing *B. vulgaris* extract (stigmasterol). Each value represents the mean ± SEM (*n* = 3).

BVEN having quercetin and stigmasterol was subjected to *in vitro* drug release by using the Franz diffusion cell. The cumulative % of drug release was calculated for BVEN. Prepared BVEN showed burst release initially followed by a steady release. Both are the main requirement for dermal application because burst release can be useful to improve drug penetration and sustained release to release drug content slowly from BVEN for long duration [50]. Cumulative % of drug release for BVEN is shown in Figures 15 and 16.

### 3.5. Drug Release Kinetics

Drug release data were explored for studying the type of release mechanism. Release kinetic study of BVEN for quercetin and stigmasterol was studied for different kinetic equations (zero-order, first-order, Higuchi, and Korsmeyer-Peppas plots). Regression coefficient (*r*^2^) values of different kinetic models for nanogels are shown in Table 8. It was found that the Higuchi plot for quercetin and zero-order plot for stigmasterol were followed in *B. vulgaris* nanogel formulation.

### 3.6. Quantitative Study

#### 3.6.1. Hair Growth Initiation

**Figure 17:**
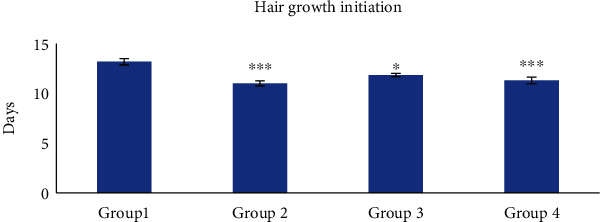
Hair growth initiation. Group 1, animals were treated with blank nanogel; group 2, animals were treated with standard (finasteride); group 3, animals were treated with 2.5% BVEN; and group 4, animals were treated with 5% BVEN. Values are expressed as mean ± SEM, in each group (*n* = 6). Data was analyzed by one-way ANOVA followed by Dunnett's *t*-test. ^∗^*p* < 0.05, ^∗∗^*p* < 0.01, and ^∗∗∗^*p* < 0.001, significance versus group 1 (vehicle).

Hair growth was started initially in group 2 and group 4 treated mice most significantly (*p* < 0.001) while group 3 animals showed less significant (*p* < 0.05) hair growth as compared to group 1 (Figure 17).

#### 3.6.2. Morphological Study

**Figure 18:**
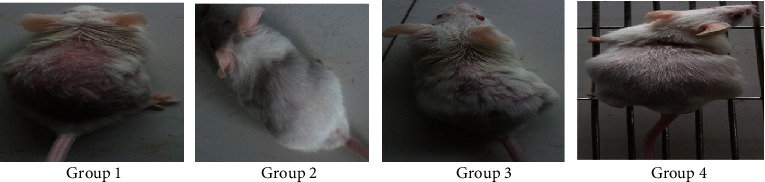
Hair regrowth at balding site on the 30th day of treatment. Group 1, animals were treated with blank nanogel; group 2, animals were treated with standard (finasteride); group 3, animals were treated with 2.5% BVEN; and group 4, animals were treated with 5% BVEN.

Hair regrowth was observed on the 30th day of treatment. Group 1 mice showed diffuse alopecia and there was no regrowth of hairs while animals in group 2 and group 4 did not show any symptoms of alopecia. Animals of group 3 showed very less hair loss. The alopecic condition was not noticeable in these groups of animals, showing that finasteride and 5% hydroalcoholic nanogel prevented the action of testosterone and inhibited testosterone-induced alopecia (Figure 18).

#### 3.6.3. Testosterone Concentration

**Figure 19:**
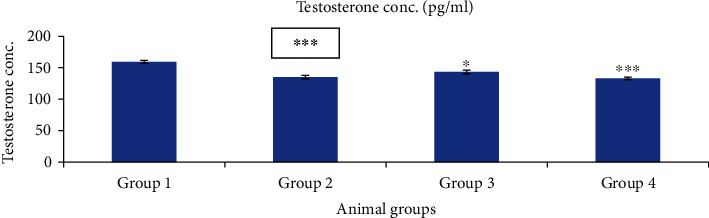
Effect of nanogels on testosterone concentration in testosterone-induced alopecia mice. Group 1, blank nanogel; group 2, standard (finasteride); group 3, 2.5% BVEN; and group 4, 5% BVEN. Values are expressed as mean ± SEM (where *n* = 6, in each group). Data was analyzed by one-way ANOVA followed by Dunnett's *t*-test. ^∗^*p* < 0.05, ^∗∗^*p* < 0.01, and ^∗∗∗^*p* < 0.001, significance versus group 1 (vehicle).

The total testosterones in scalp are produced by androgen hormones. Testosterone is converted into dihydrotestosterone by the enzyme 5-*α* reductase-II. Increased levels of dihydrotestosterone bind to androgen receptors and reduced blood supply in the scalp; consequentially, hair growth get arrested. There was most significant (*p* < 0.001) decrement in testosterone levels in group 2 and group 4 treated mice as compared to group 1 (control). Group 3 mice showed less significant (*p* < 0.05) decrease in testosterone level as compared to group 1, shown in Figure 19.

#### 3.6.4. Total Protein Content

**Figure 20:**
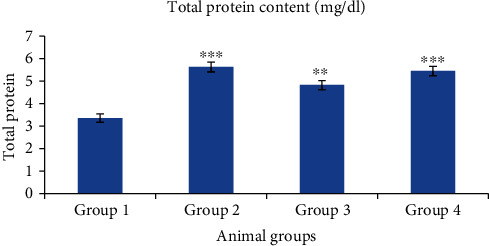
Effect of nanogels on total protein content in testosterone-induced alopecia mice. Group 1, blank nanogel; group 2, standard (finasteride); group 3, 2.5% BVEN; and group 4, 5% BVEN. Values are expressed as mean ± SEM (where *n* = 6, in each group). Data was analyzed by one-way ANOVA followed by Dunnett's *t*-test. ^∗^*p* < 0.05, ^∗∗^*p* < 0.01, and ^∗∗∗^*p* < 0.001, significance versus group 1 (vehicle).

It was revealed that total protein content of group 2 and group 4 treated mice showed most significant increase (*p* < 0.001) as compared to group 1. Group 3 mice showed significant (*p* < 0.01) increase in total protein content (Figure 20). Increased level of proteins might increase the growth factors or signalling proteins responsible for hair growth (*β*-FGF, KGF, PDGF-AA, and VEGF) [51].

#### 3.6.5. Prostate Gland Weight

Animal study showed insignificant changes in prostate gland weight shown in Table 9, which indicates that topical doses were insufficient to inhibit 5-alpha reductase-II in the prostate gland and only in the skin dihydrotestosterone inhibition was accomplished [52].

### 3.7. Quantitative Study

#### 3.7.1. Hair Follicular Density

**Figure 21:**
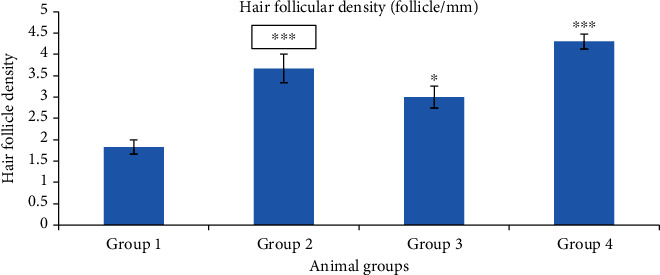
Hair follicular density. Group 1, blank nanogel; group 2, standard (finasteride); group 3, 2.5% BVEN; and group 4, 5% BVEN. Values are expressed as mean ± SEM (where *n* = 6, in each group). Data was analyzed by one-way ANOVA followed by Dunnett's *t*-test. ^∗^*p* < 0.05, ^∗∗^*p* < 0.01, and ^∗∗∗^*p* < 0.001, significance versus group 1 (vehicle).

Hair follicle density is defined as number of hair follicle per mm. It is depicted from histological study that hair density had increased most significantly (*p* < 0.001) in groups 2 and 4 compared to group 1 (Figure 21). But in the case of group 3, it increased less significantly.

#### 3.7.2. Anagen/Telogen Ratio

By using ocular micrometre, no. of hair follicles present in anagen and telogen phase was counted in 1 mm area (Table 10). Animals of groups 2 and 4 showed most significant (*p* < 0.001) A/T ratio as compared to group 1 (control). Group 3 showed significant anagen/telogen ratio.

#### 3.7.3. Histopathology of Mice Skin

**Figure 22:**
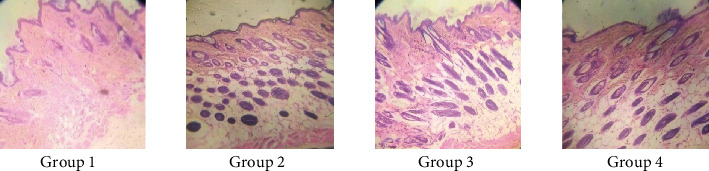
Histopathological changes on mouse skin photomicrographs (10x) of H&E stained 20th day. Group 1, control nanogel; group 2, standard (finasteride); group 3, 2.5% nanogel of hydroalcoholic extracts of *B. vulgaris*; and group 4, 5% nanogel of hydroalcoholic extracts of *B. vulgaris.*

**Figure 23:**
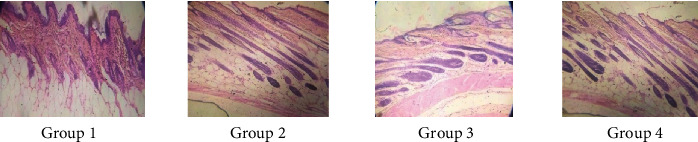
Histopathological changes on mouse skin photomicrographs (10x) of H&E stained 30th day. Group 1, control nanogel; group 2, standard (finasteride); group 3, 2.5% nanogel of hydroalcoholic extracts of *B. vulgaris*; and group 4, 5% nanogel of hydroalcoholic extracts of *B. vulgaris.*

Androgenetic alopecia in men is developed by testosterone, along with a genetic predisposition [44, 53, 54]. Testosterone and dihydrotestosterone promote hair follicle shrinkage, according to histopathology of skin sections [55]. Alopecia was induced in mice of all groups by administration of testosterone. Skin samples of experimental mice were taken for histopathological study after 20th and 30th days of treatment. In group 1 mice, hair follicles were in the telogen phase, seemed bulbous, and were small and uneven (Figures 22 and 23, group 1). All other groups were compared with group 1. Simultaneous administration of finasteride (standard) and hydroalcoholic nanogel containing 2.5% extract or 5% extract (group 2, group 3, and group 4, respectively) blocked the action of testosterone; thus, alopecia was not observed. There was an increase in the number of hair follicles, and miniaturization of hair follicle was inhibited by administration of nanogel containing hydroalcoholic extracts and standard drug finasteride. During the anagen phase follicles were longer, deeper, and denser, cell necrosis was lesser, and uniform distribution of melanin content was more after 30 days as compared to 20-day treatment. The studies of hydroalcoholic extracts of *B. vulgaris* in our laboratory revealed the presence of stigmasterol and quercetin along with other phytoconstituents. Quercetin and stigmasterol have been shown to inhibit the production of DHT from testosterone by blocking the action of 5-*α*-reductase inhibitory and decreasing oxidative stress [51, 56].

Testosterone-induced alopecia involves the progressive hair loss, in response to circulating androgens [57]. Till date, only two FDA-approved “hair loss drugs” finasteride (5-*α*-reductase inhibitor) and minoxidil (antihypertensive potassium channel opener) are supremely used in the current clinical practice [58], but adverse reactions, like itching and dermatitis, have been reported. Hence, the drugs from plant origin can be used to replace the synthetic one to decrease the side effects.

Nanotechnology is a novel field that utilizes therapeutically active nanomaterials having unique physicochemical features for targeting it into the hair follicles [59]. Hair follicle is regarded as the most efficacious route especially when the drug is incorporated into nanocarriers as it leads to enhanced drug penetration and deeper release in the skin stratum corneum [60]. For treatment of alopecia, it is important to accumulate drug in the follicular unit. When treatments are targeted, this potentiates drug bioavailability leading to desired effect and also small-drug concentrations are needed [61]. By establishing stable and homogeneous systems that are released in a predictable manner, the use of various polymers for drug loading and delivery in hair follicles represents a powerful noninvasive method of administration [62]. BVEN formulation was sustained release in nature; thus, it was prepared for the treatment of testosterone-induced alopecia. From biological evaluation, it was found that prepared 5% hydroalcoholic extract nanogel showed most significant hair growth initiation, decreased testosterone levels, increased total protein content, increased anagen/telogen ratio, and increased hair follicular density as compared to control group, while 2.5% treated hydroalcoholic extract nanogel animals showed significant increased anagen/telogen ratio, increased total protein content but less significant hair growth initiation, decreased testosterone levels, and increased hair follicular density as compared to control group which indicates that 5% hydroalcoholic extract nanogel has more potential to treat testosterone-induced alopecia.

## 4. Conclusion

The optimized nanoparticles had an entrapment efficiency of 82% and 79% for quercetin and stigmasterol, respectively, and particle size of 274 nm as confirmed by zeta sizer method. Transmission electron microscopy showed 250-280 nm sized and spherical-shaped nanoparticles. FTIR confirmed the presence of stable nanoparticle which was stable through electrostatic bond. Nanogel pH 6.5, drug content, i.e., quercetin 99.84 ± 1.30% and stigmasterol 99.89 ± 1.52%, spreadability 20.3 ± 0.5925 g cm/second, and viscosity 110 × 10^5^ cps were observed. Stability studies showed that nanogel was stable at 4°C ± 2°C/60% ± 5% RH. *In vitro* drug release study confirmed that nanogel was sustained release. The Higuchi plot for quercetin and zero-order plot for stigmasterol was followed in BVEN. 5% BVEN showed promising results for the treatment of testosterone-induced alopecia which is the main goal of our study; increased deeper penetration of phytoconstituents in the skin makes them potential candidate for management of testosterone-induced alopecia.

## Figures and Tables

**(a):** 

Formulation	Sodium alginate	Calcium chloride
F1	0	0
F2	0	0
F3	0	0
F4	0	0
F5	0	+1
F6	+1	+1
F7	−1	0
F8	0	−1
F9	+1	−1
F10	0	0
F11	+1	0
F12	−1	−1
F13	−1	+1

**(b):** 

Coded values	Actual values	
	Sodium alginate (mg)	Calcium chloride (mg)
-1	0.5	0.1
0	1.0	0.3
+1	1.5	0.5

**Table 2:** Central composite design for nanoparticles containing *B. vulgaris* leaf extract for quercetin.

Run	Factor 1 (sodium alginate in mg/ml)	Factor 2 (calcium chloride in mg/ml)	Response 1 (entrapment efficiency in %)	Response 2 (particle size in nm)
1	+1	0	56	549.7
2	0	-1	62	405
3	-1	0	71	517
4	+1	+1	82	247
5	-1	-1	85	509.8
6	0	0	61	472.1
7	-1	+1	69	674
8	0	+1	73	434.6
9	0	0	60	405
10	+1	-1	64	712
11	0	0	65	478.5
12	0	0	67	476
13	0	0	61	428.7

**Table 3:** Central composite design for nanoparticles containing *B. vulgaris* hydroalcoholic extract for stigmasterol.

Std	Run	Factor 1 A (sodium alginate in mg/ml)	Factor 2 B (calcium chloride in mg/ml)	Response 1 (entrapment efficiency in %)	Response 2 (particle size in nm)
11	1	0	0	69	476
7	2	0	-1	49	405
6	3	+1	0	60	549.7
12	4	0	0	67	428.7
10	5	0	0	69	472.1
9	6	0	0	59	478.5
3	7	-1	+1	68	674
2	8	+1	-1	60	712
4	9	+1	+1	79	247
13	10	0	0	60	405
5	11	-1	0	43	517
1	12	-1	-1	55	509.8
8	13	0	+1	72	434.6

**Table 4:** Evaluation parameters for nanogel formulation.

S. no.	Nanogel	Physical appearance	pH	Spreadability (g cm/sec)	Viscosity (cps)
1	Control nanogel	White, smooth, clear, and transparent	6.2	22.9 ± 0.4583	95.4 × 10^5^
2	*Beta vulgaris*	Light green, smooth, clear, and translucent	6.5	20.3 ± 0.5925	110 × 10^5^

**Table 5:** Stability studies at 4°C ± 2°C/60% ± 5% RH for 3 months.

S. no.	Nanogel	Physical appearance	pH	Drug content (%)		Spreadability (g cm/sec)	Viscosity (cps)
				Quercetin	Stigmasterol		
1	Control nanogel	White, smooth, clear, and transparent	6.3	—		21.6 ± 0.8819	95 × 10^5^
2	*Beta vulgaris*	Light green, smooth, clear, and translucent	6.5			19.6 ± 0.33	110 × 10^5^
				99.25 ± 1.8	99.53 ± 1.5		

**Table 6:** Stability studies at 25°C ± 2°C/60% ± 5% RH for 3 months.

S. no.	Nanogel	Physical appearance	pH	Drug content (%)		Spreadability (g cm/sec)	Viscosity (cps)
				Quercetin	Stigmasterol		
1	Control nanogel	White, smooth, clear, and transparent	6.8	—		20 ± 0.5774	98 × 10^5^
2	*Beta vulgaris*	Light green, smooth, clear, and translucent	6.9	93.20 ± 1.5	91.03 ± 1.2	18.3 ± 0.33	109 × 10^5^

**Table 7:** Stability studies at 40°C ± 2°C/60% ± 5% RH for 3 months.

S. no.	Nanogel	Physical appearance	pH	Drug content (%)		Spreadability (g cm/sec)	Viscosity (cps)
				Quercetin	Stigmasterol		
1	Control nanogel	White, smooth, clear, and transparent	6.3			13.3 ± 0.8819	98.4 × 10^5^
2	*Beta vulgaris*	Light green, smooth, clear, and translucent	7.0	84.63 ± 1.32	86.30 ± 0.63	14.6 ± 0.333	128 × 10^5^

**Table 8:** *R* ^2^ value of different plot for *B. vulgaris* extract nanogel.

Plot	Quercetin	Stigmasterol
Zero-order plot	0.973	0.9845
First-order plot	0.8886	0.8055
Higuchi plot	0.9926	0.9262
Korsmeyer-Peppas plot	0.9782	0.9269

**Table 9:** Prostate gland weight measurement of treated animal group.

Group	Prostate gland weight
Group 1	0.0383 ± 0.0004
Group 2	0.0328 ± 0.0004
Group 3	0.0358 ± 0.002
Group 4	0.033 ± 0.0005

Group 1 (control), animals were treated with blank nanogel; group 2, animals were treated with standard (finasteride); group 3, animals were treated with 2.5% BVEN; and group 4, animals were treated with 5% BVEN. Values are expressed as mean ± SEM (where *n* = 6, in each group).

**Table 10:** A/T ratio in skin sections of different groups of animals.

Group	A/T ratio
Group 1	0.5 ± 0.098
Group 2	4 ± 0.000^∗∗∗^
Group 3	2.8 ± 0.1453^∗∗^
Group 4	4.16 ± 0.4410^∗∗∗^

Group 1, control nanogel; group 2, standard (finasteride); group 3, 2.5% of BVEN; and group 4, 5% nanogel of BVEN. Values are expressed as mean ± SEM (where *n* = 6, in each group). Data was analyzed by one-way ANOVA followed by Dunnett's *t*-test. ^∗^*p* < 0.05, ^∗∗^*p* < 0.01, and ^∗∗∗^*p* < 0.001, significance versus group 1 (vehicle).

## Data Availability

Data are available within the manuscript.
